# Supplementary figures and images for: Population Movement and Poliovirus Spread across Pakistan and Afghanistan in 2023
Source: Vaccines (Basel). 2024 Sep 1;12(9):1006. doi: 10.3390/vaccines12091006 (PMC11435525; doi:10.3390/vaccines12091006)

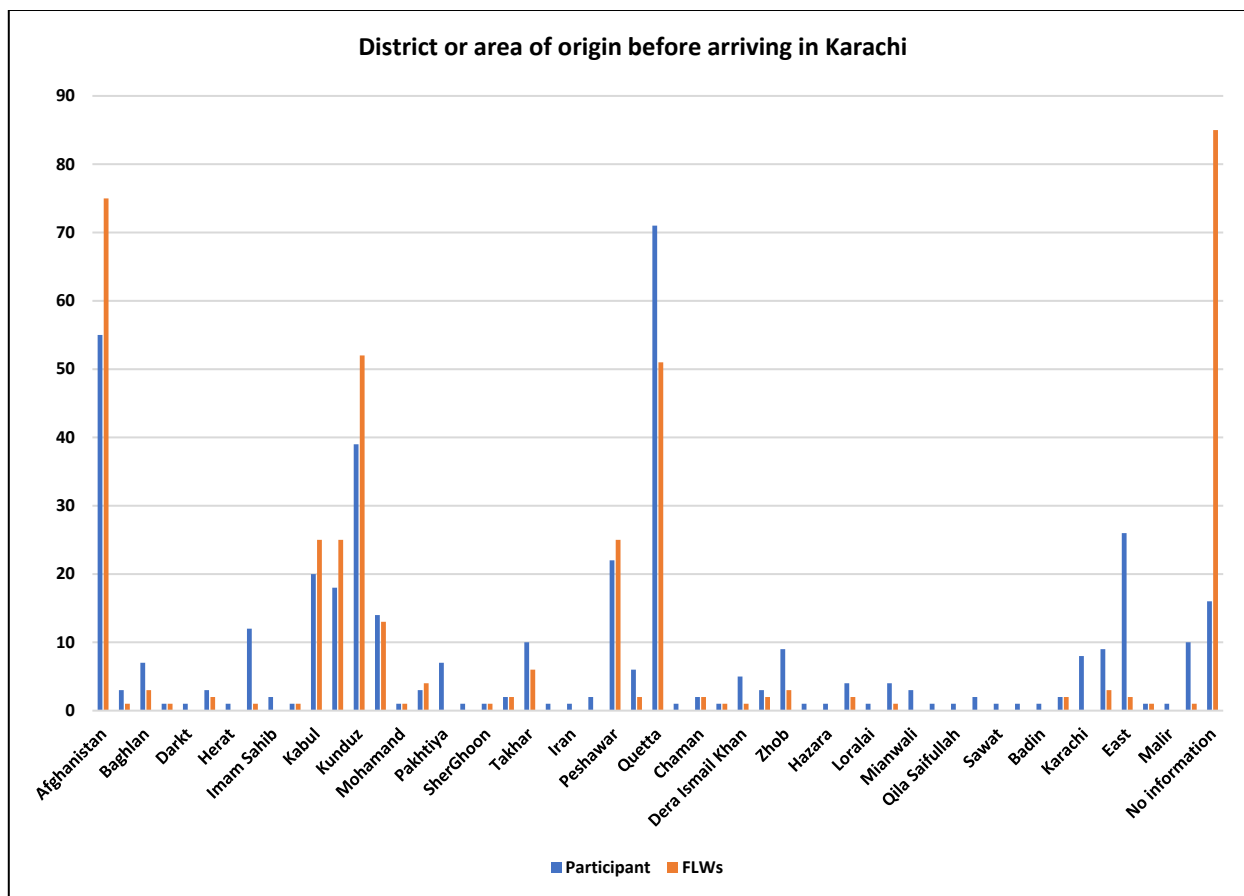

Figure S1: Origin of Afghan population before arriving in Karachi, Sindh province, Pakistan.

Supplement: Supplementary file 1 [file vaccines-12-01006-s001.zip › vaccines-3087882-supplementary.pdf]
